# High Dose Steroid Therapy to Prevent Severe Hypoxia in COVID-19 Patients: A Potential Solution for Low Resource Clinical Setting

**DOI:** 10.7759/cureus.12330

**Published:** 2020-12-27

**Authors:** Lokesh Edara, Tarun Kumar Suvvari, Lakshmi Venkata Simhachalam Kutikuppala

**Affiliations:** 1 Internal Medicine, Western Michigan University, Kalamazoo, USA; 2 Medicine, Rangaraya Medical College, Kakinada, IND; 3 Medicine, Konaseema Institute of Medical Sciences and Research Foundation (KIMS&RF), Amalapuram, IND

**Keywords:** acute respiratory distress syndrome (ards), high dose corticosteroids, covid 19, hypoxia, sars cov-2

## Abstract

Coronavirus disease 2019 (COVID-19) can lead to severe respiratory failure; about 5%-10% of patients progress to severe pneumonia and respiratory distress, leading to multi-system failure. Dexamethasone helped to prevent mortality in COVID-19 patients. Low resource population in developing countries has limited access to critical care, but they do have access to oral and IV corticosteroids, anti-hyperglycemic agents, and anticoagulants. We report two patients with severe COVID-19 successfully treated with a high dose of methylprednisolone therapy. Early intervention with high dose corticosteroids in COVID-19 patients could be a solution for pacifying cytokine storms and reducing morbidity and mortality.

## Introduction

In developing countries, there are limited resources for patients with coronavirus disease 2019 (COVID-19). They do not have access to critical care facilities, ventilators, oxygen, sophisticated medical services, and supportive care. They have access to relatively inexpensive medications such as steroids, insulin, oral diabetic medications, and anticoagulants. As of late November 2020, severe acute respiratory syndrome coronavirus 2 (SARS-CoV-2) has infected more than 51 million people worldwide, with more than 1.2 million deaths [1]. Even though most of the patients were expected to recover, there is high morbidity and mortality in certain populations, including the elderly and men [2]. Dexamethasone is shown to reduce mortality in COVID-19 patients [3-4]. High dose steroids were given in several clinical conditions such as systemic lupus erythematosus, acute renal allograft rejection, juvenile rheumatoid arthritis, juvenile dermatomyositis, pemphigus, optic neuritis, multiple sclerosis, acute disseminated encephalomyelitis, giant cell arteritis, and multiple sclerosis. The common denominator in all these conditions is severe acute inflammation. Pulse dose steroids (such as methyl prednisolone with doses as high as 1 g) are used to subdue cytokine storm, thereby reducing morbidity and mortality in these conditions with severe inflammation. As COVID-19 infection induces severe multi-system inflammation, leading to increased morbidity and mortality, it is plausible that high dose steroids could be used in this condition.

Here we describe two patients with severe COVID-19 infection successfully treated with a high dose of steroids methylprednisolone therapy in elderly males. These early and high dose corticosteroids in COVID-19 patients could be a solution for pacifying cytokine storms in both developed and developing low resource population. 

## Case presentation

Patient 1

**Figure 1:**
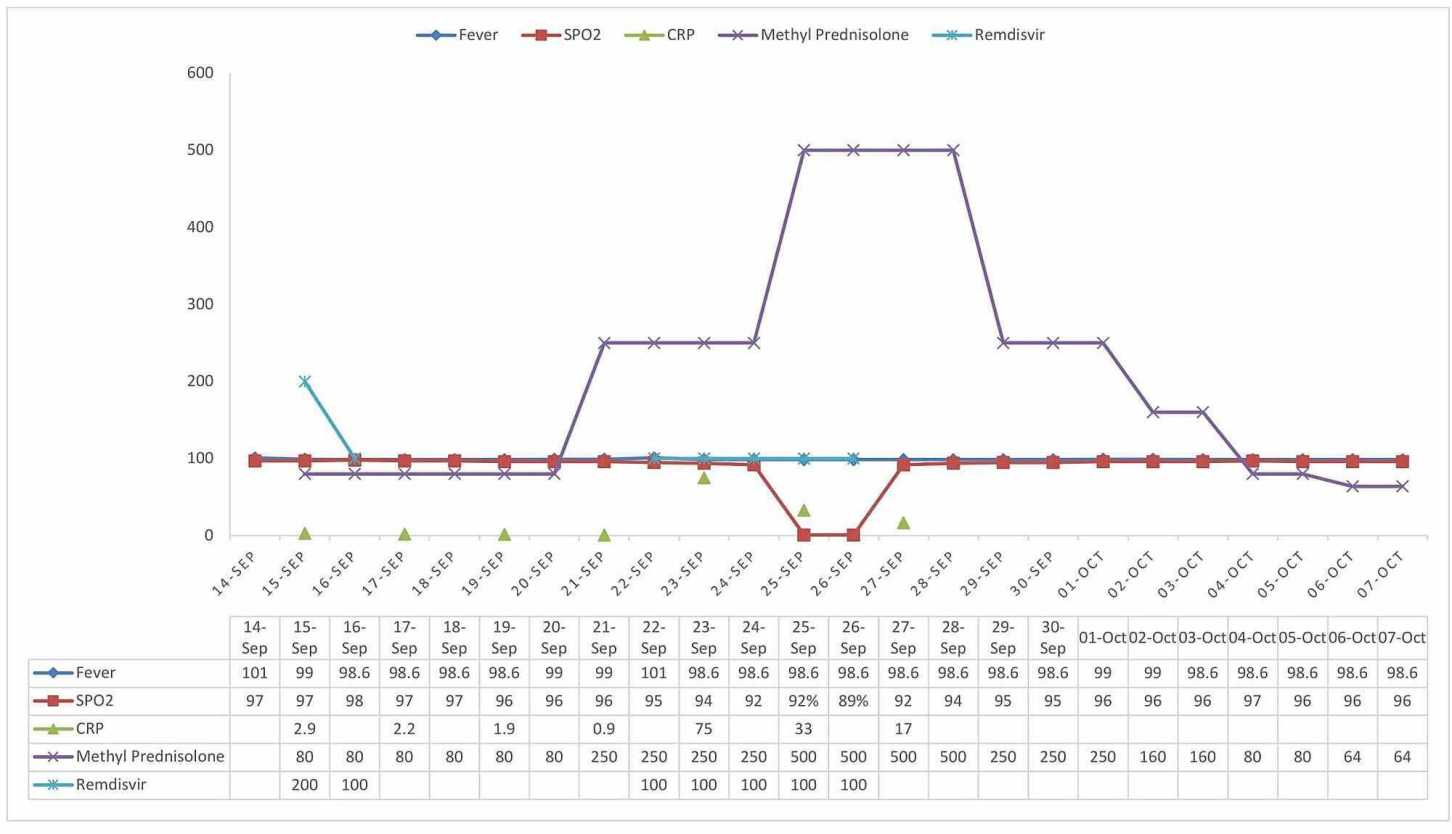
Graphical representation of some vital trends of the first case. Measurement: Fever - ℉, SPO2 - %, C-reactive protein (CRP) – mg/L, Methyl prednisolone – mg/day, Remdesivir – mg/dose

A healthy 63-year-old male, history of presented with cough, fever, extreme weakness of two-day duration. His rapid antigen test for COVID-19 was positive. He was started on ivermectin, doxycycline, and rivaroxaban. Paracetamol was used for symptomatic treatment for fever, and the fever subsided on day 3. On day 5, the fever recurred. He was started on methylprednisolone 40 mg twice a day and continued till day 12. As the patient developed mild hypoxia (O2 sats dropped to 92%) and dyspnea, he was admitted to the hospital on day 12 and 2 L of oxygen were given through continuous positive airway pressure (CPAP). He received one dose of remdesivir, convalescent plasma, and anticoagulant was switched to enoxaparin 60 mg twice a day. His methylprednisolone dose was increased to 125 mg twice a day on day 13. As his C-reactive protein (CRP) levels increased to 80.2, and as the patient is still hypoxic (O2 saturation in high 80s), to subdue cytokine storm, methylprednisolone dose was increased to 250 mg three times a day on day 14 and continued until day 22. His CRP levels improved to 3.1 on day 22, so does his clinical condition. His ferritin levels fluctuated from 199 to 715 units (highest on day 21 and subsequently returned to normal). We preferred methylprednisolone over decadron as it has higher lung tissue penetration and methylprednisolone was slowly reduced in another week. As the patient's clinical condition improved, he was discharged home on day 30 on a tapering dose of steroids. The graphical representation of some vital trends of the first case was described in Figure 1.

Patient 2

**Figure 2:**
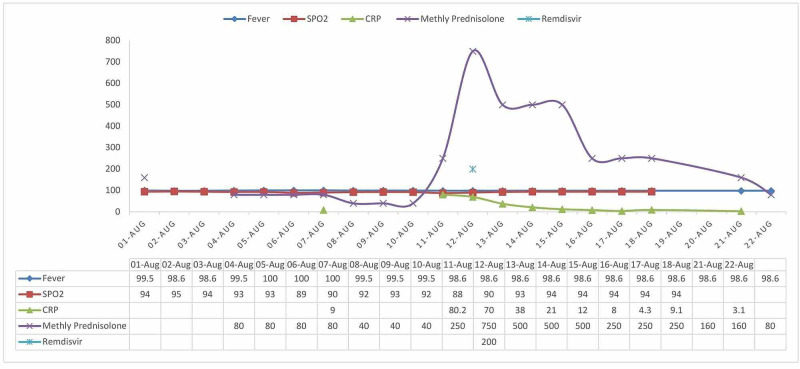
Graphical representation of some vital trends of the second case. Measurement: Fever - ℉, SPO2 - %, C-reactive protein (CRP) – mg/L, Methyl prednisolone – mg/day, Remdesivir – mg/dose

Another 76-year-old male with a past medical history of non-insulin-dependent diabetes mellitus, coronary artery disease with coronary stents, and bilateral knee replacement presented with fever and generalized malaise. Rapid antigen test for COVID-19 was positive. He was admitted to the hospital on day 1, and symptomatic treatment was given for high fever (101°F). Remdisivir was given on day 2 (200 mg) and day 3 (100 mg). The patient was also started on methylprednisolone 40 mg twice daily from day 2. His fever improved promptly and bradycardia was seen. On day 9, his fever spiked again. His methylprednisolone dose was increased to 125 mg twice a day. Remdisivir was also restarted and received two doses (days 9 and 10). As the patient was still symptomatic (hypoxia and fever) and there was a rapid increase in CRP levels, methylprednisolone dose was increased to 250 mg twice a day on day 12. Methylprednisolone was tapered gradually to 64 mg per day on day 23. As his clinical condition improved significantly, the patient was discharged home on day 24. His CRP levels came to normal in a few days. The graphical representation of some vital trends of the second case was described in Figure 2.

## Discussion

COVID-19 viral infection is a pandemic all over the world. Even though most of the COVID-19 infected patients (80%) survive and incur mild symptoms, a severe infection could develop in about 10%-20%. The mortality rate could be about 2%, but it may increase with age [2]. COVID-19 can affect anyone. But, older people and those with underlying conditions such as diabetes mellitus, hypertension, cardiorespiratory disorders, chronic liver, and renal diseases could develop severe infection with complications and even death [5]. Patients with severe COVID-19 develop multi-organ failure secondary to pro-inflammatory cytokine storm. These cytokines include but not limited to interleukin (IL)-6, IL-10 and tumor necrosis factor α, granulocyte colony-stimulating factor, monocyte chemoattractant protein 1, macrophage inflammatory protein 1α, and increased expression of programmed cell death 1, T-cell immunoglobulin and mucin domain 3 (Tim-3) [6]. The cytokines may inflame several organs, including lung (pulmonary infiltrates and hypoxia), heart and blood vessels (diffuse microangiopathic thrombi, myocarditis, acute coronary syndrome, cardiac arrhythmias, and heart failure), gastrointestinal system (diarrhea, nausea, vomiting, and abdominal pain), blood clotting system (disseminated intravascular coagulation, venous thromboembolism) [7]. Apart from prevention measures such as face coverings and social distancing, the management of infected patients is symptomatic. Several pharmacological and nonpharmacological agents were shown to be effective in reducing morbidity and mortality. These include steroids such as dexamethasone [3-4], low dose methylprednisolone [8], antiviral agents such as remdesivir [9], immunomodulatory antibodies such as tocilizumab [10], and convalescent plasma [11]. Recently, Pfizer and Moderna pharmaceutical companies announced that their mRNA vaccines are effective in about 95% of people in preventing COVID-19 infection. Despite this announcement, it may take several months to a year for global vaccination to complete [12]. Meanwhile, treatment with anti-inflammatory agents is very important in saving the lives of symptomatic COVID-19 patients.

Despite these agents' development, mortality rates in developing countries with low resources are high, as there is limited availability of the agents mentioned above and/or sophisticated hospital and intensive care facilities with ventilators. But in these countries, there is the availability of cheaper options such as steroids, insulin to control hyperglycemia, antibiotics to control superinfections, and pneumonia. Early intervention with steroids was shown to improve morbidity and mortality in COVID-19 patients, as shown by Recovery study investigators in England [3] and low dose methylprednisolone in Michigan [8]. 

But early intervention with high dose steroids (high dose methylprednisolone) is not reported or studied in COVID-19 patients. Here we report two patients who received high dose steroids early in their disease course and survived the severe COVID-19 infection. Interestingly, our two patients who received high dose steroids did not require high flow oxygen, ventilator, and ICU admission. In clinical practice, high dose steroids are routinely used in several situations such as sepsis (stress dose steroids), lupus nephritis, scleroderma renal crisis, vasculitis, severe asthma, and allergic conditions. In children, high dose methylprednisolone (2 mg/kg) is being used to treat the multi-system inflammatory syndrome [13]. So, we hypothesized that high dose steroids in the form of high dose methylprednisolone, with doses more than 250 mg/day, could subdue the cytokine storm in our patients and reduce morbidity and mortality.

Both of our patients were treated with low dose steroids, remdesivir. Since their clinical condition did not improve with these measures, we decided to increase the dose of methylprednisolone and went up to doses as high as 500-750 mg/day for several days and then slowly weaned them off of steroids over several days. Recently, So et al. treated COVID-19 patients intubated secondary to acute respiratory distress syndrome with high dose methylprednisolone 500-1000 mg/day for a short time followed by a taper. They reported a positive outcome in all seven patients [14]. High dose steroids have been largely discouraged as they cause immunosuppression and thereby worsening viral propagation. As COVID-19 induces severe inflammatory response and cytokine storm, short course high dose steroids would subdue that inflammation and decrease the morbidity and mortality [15].

## Conclusions

Our experience of these two patients, treated with high dose steroids, leads to severe COVID-19 infection survival. They did not require much oxygenation, not needed a ventilator and ICU admission. This treatment option could be very useful to save lives in low resource countries. Even in highly resourceful developed countries, early intervention with high dose steroids could reduce cost and more invasive interventions such as intubation, ventilator support, and ICU admission. But we need well-designed research studies in the near future.
